# Association of personalized dietary advice aiming to increase protein intake with macronutrient intake of community-dwelling older adults: a secondary analysis of the PROMISS RCT

**DOI:** 10.1007/s41999-025-01267-z

**Published:** 2025-07-01

**Authors:** Riikka T. Niskanen, Hanneke A. H. Wijnhoven, Kaisu H. Pitkälä, Marjolein Visser, Hannu Kautiainen, Merja H. Suominen, Satu K. Jyväkorpi

**Affiliations:** 1 Unit of Primary Health Care, University of Helsinki, Department of General Practice and Primary Health Care, and Helsinki University Central Hospital, Tukholmankatu 8, 00014 Helsinki, Finland; 2 Department of Health Sciences, Faculty of Science, and the Amsterdam Public Health Research Institute, Vrije Universiteit Amsterdam, Amsterdam, The Netherlands

**Keywords:** Dietary intervention, Nutrient intake, Older adults, Community-dwelling older persons

## Abstract

**Purpose:**

The PROMISS randomized-controlled trial (RCT) showed that personalized dietary advice, regarding, for instance, protein-enriched food products, increased protein intake among community-dwelling older adults with a low habitual protein intake. This secondary analysis evaluates how the dietary advice aimed solely at increasing protein intake affects the intake of other (macro)nutrients, saturated fat, sugars, and dietary fiber.

**Methods:**

Community-dwelling older adults (*n* = 260, mean age 75 years, 53% women) in Finland and the Netherlands with a habitual protein intake < 1.0 g/kg adjusted body weight/day were included in this secondary analysis. The two intervention groups (*n* = 175) received personalized dietary advice for 6 months to increase protein intake. The control group (*n* = 85) received no intervention. Nutrient intake was evaluated with 24 h recalls and a 3-day food record at baseline and follow-up.

**Results:**

Macronutrient intake at baseline was similar across groups. In addition to an increase in protein, the intervention groups showed a** s**ignificant increase in carbohydrate intake relative to the control group, with no change in the intake of total fat, saturated fat, sugars, or dietary fiber. An increase in energy intake was associated with an increase in protein intake [*r* = .55 (95% CI 0.46–0.63)] but not with relative weight gain [*r* = 0.03 (95% CI − 0.09 to 0.16)].

**Conclusion:**

Dietary advice to increase protein intake increased also the intake of carbohydrates, but intakes of fiber, sugars, and (saturated) fat remained at baseline levels.

## Key summary points

**Aim:**

This secondary analysis aimed to study how dietary advice focusing on protein intake impacts the intake of other (macro) nutrients and further explored how changes in macronutrient intakes and body weight were associated with observed increase in energy intake.

**Findings:**

This study showed a significant increase in carbohydrate intake relative to the control group, with no change in the intake of total fat, saturated fat, sugars, or dietary fiber. An increase in energy intake was associated with an increase in protein intake but not with relative weight gain.

**Message:**

Dietary advice to increase protein intake affects intake of carbohydrates and energy alongside the increase in protein intake among community-dwelling older adults with a low protein intake.

## Introduction

Malnutrition, especially low protein intake, threatens physical functioning and independent living in the older persons. Low protein intake has been associated with lower muscle mass and strength, leading to such negative health consequences as sarcopenia [1], falls [2], and frailty [3]. Estimated 70% of older community-dwelling adults do not meet the protein intake of 1.0–1.2 g/kg body weight (BW)/d currently recommended by the scientific literature [4–7].

Nutrition interventions, including personalized dietary advice, have been successful in increasing protein intake through diet [8–10]. Many of these interventions [8, 9] have considered the overall diet quality, providing multiple goals with advice. However, when considering the nutritional problems among older community-dwelling adults, e.g., protein–energy malnutrition [11], personalized dietary advice focusing only on the intake of protein might be an effective way of improving physical functioning through diet. Nevertheless, the dietary advice should not deteriorate the overall diet quality at the expense of increasing protein intake.

Also increasing rates of obesity threaten the physical functioning of older adults [12]. Older adults with a low protein diet, sarcopenia, or frailty may also have obesity [13, 14]. Research has shown that sarcopenia and obesity together impose a double burden on physical functioning [15, 16]. In addition, lower protein intake is found to be more prevalent among those with a higher body mass index (BMI) [4]. Hence, increasing protein intake should be achieved without excess weight gain to avoid increasing the risk of non-communicable diseases.

The PRevention Of Malnutrition In Senior Subjects in the EU (PROMISS) randomized-controlled trial (RCT) [17] is aimed at prevention of malnutrition in European older adults through personalized dietary advice, including recommendations for protein-enriched food products. The 6-month intervention among community-dwelling older adults with low protein intake resulted in a significant increase in protein intake and improvement in physical functioning measured by 400-m walk time (primary outcome) and leg extension strength [18]. The intervention aimed at energy-neutral advice to avoid (excess) weight gain; however, the participants made independent decisions on food intake on a daily basis. Nevertheless, there was no change in body weight (measured in kg) despite a reported increase in energy intake [18]. The effect of the dietary advice on the intake of other (macro)nutrients was not reported.

The model of the dietary advice applied in the PROMISS RCT could be applicable for increasing protein intake among community-dwelling older adults. The dietary advice was found to be feasible and well received by participants [19]. However, it is not known how the advice focusing on protein intake impacts the intake of other nutrients, which is an important aspect when considering overall health and the risk of non-communicable diseases. Hence, this secondary analysis of the PROMISS RCT evaluates the changes in macronutrient intake and the intakes of saturated fat, sugars, and dietary fiber. We also further explored how changes in macronutrient intakes and body weight were associated with observed increase in energy intake.

## Methods

### Summary of the PROMISS study design

Details of the PROMISS RCT design [17], the study intervention, and the primary results [18] have been reported previously. Briefly, PROMISS RCT was a multicenter randomized-controlled trial conducted in Finland and The Netherlands between November 2018 and July 2020 (ClinicalTrials.gov identifier: NCT03712306). Community-dwelling adults aged ≥ 65 years who had a protein intake < 1.0 g/kg adjusted body weight (aBW)/d and were able to walk independently were recruited for a 6-month intervention. This RCT included three study groups of which two received personalized dietary advice to increase protein intake from the diet and one was the control group.

Ethical approval was provided by the Ethics Committee of Helsinki University Central Hospital, Finland (HUS/1530/2018) and the Medical Ethics Committee of Amsterdam UMC, location VUmc, Amsterdam, The Netherlands (2018.399). Oral informed consent was obtained from participants before the screening procedure and written informed consent at the start of the first clinical visit.

### Study participants

A total of 276 participants were recruited to the PROMISS RCT. The main exclusion criteria were protein intake ≥ 1.0 g/kg aBW/d, BMI < 18.5 or > 32 kg/m^2^, purposeful weight loss or gain > 3 kg over the past 3 months, weekly use of alcohol in high doses, severe food allergy or a vegan diet, diagnosis of an eating disorder or another medical condition requiring dietary treatment, and Mini-Mental State Examination (MMSE) score ≤ 20.

**Fig. 1:**
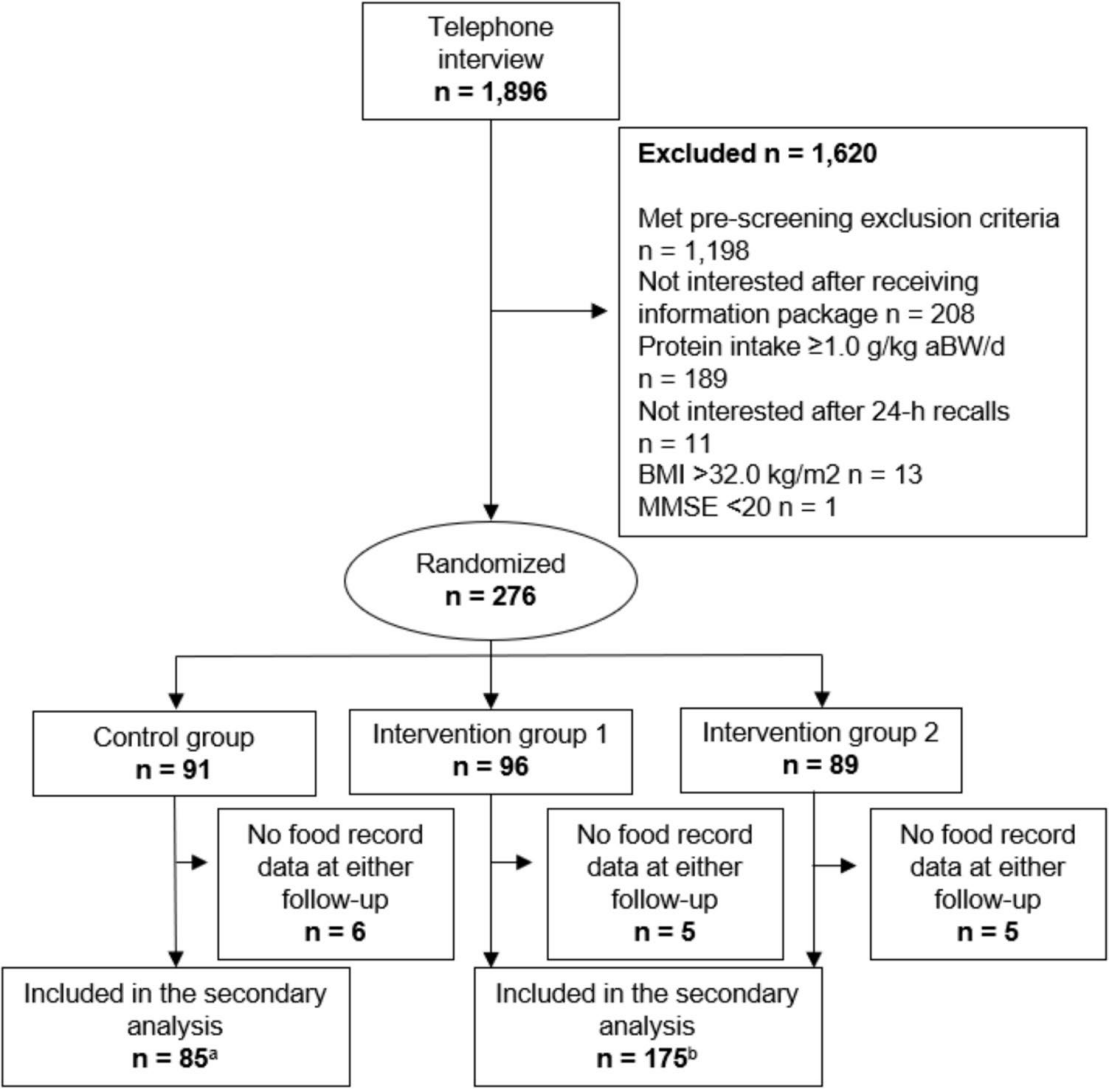
Flowchart of the inclusion of study participants in the secondary analysis. ^a^Lack of one food record at 6-month follow-up; ^b^lack of two food records at 6-month follow-up

For this secondary analysis, we included all participants who had provided food record data at either the 3-month (*n* = 3) or 6-month (*n* = 257) follow-up. Hence, 260 participants (53% women) were included. The two intervention groups receiving dietary advice were analyzed as one group, since no differences between the groups emerged in the primary results [18]. A flowchart of the selection of participants is presented in Fig. 1.

### Dietary advice

A detailed description of the content and tools of the dietary advice has been reported elsewhere [19]. In short, the dietary advice consisted of one 60-min individual advice meeting at the beginning of the trial and additional advice at the 3-month follow-up, if needed. The participants in both intervention groups were advised to increase their daily protein intake to at least 1.2 g/kg aBW/d (energy-neutral) and to include each day at least one high-protein meal with ≥ 35 g of protein. In addition, intervention group 2 was advised to time protein intake within half an hour after usual physical activity.

The nutritionists used several tools to support the participants in following the dietary advice. The nutritionist prepared a sample menu for 1 day that followed the participant’s usual meal pattern and preferences. The participant was encouraged to vary the menu as long as the target amount of protein was met and at least one high-protein meal was included. The nutritionists provided general information about protein and its sources to the participant and demonstrated how protein content information could be found from product labels. Participants received a booklet with written instructions on the diet. They were also provided with protein-enriched food products (e.g., puddings, protein bar, whole‐grain cereals, whey protein powder, and protein drink) free of charge. Only types of products that participants already consumed in their regular diet were offered to avoid potential deterioration of the quality of the diet. Energy-dense foods were advised to be eaten no more often than currently to avoid excess weight gain. The control group received a general leaflet on nutrition, and they were advised not to make any changes to their dietary habits or physical activity level during the study period.

All participants received a monthly evaluation call from the nutritionists. The participants in the intervention groups received additional advice on how to adapt the sample menu and find protein-rich alternatives. For the control group, the call was to ask how they were doing.

### Measures

The baseline measures included demographics (age, sex, education, and household), BMI, body composition, mobility, functional ability, self-rated health, number of medications, and smoking history. BMI was calculated based on measured weight and height at the baseline visit. In addition, body composition was measured with bioelectrical impedance using the BodyStat 1500MDD device. Body fat percentage (%) was calculated using the Kyle equation [20]. Mobility was evaluated with a question about difficulties in walking, which is part of the EuroQol 5D-5L questionnaire [21] evaluating quality of life. Responses “I have no problems with walking” and “I have some problems with walking” were perceived as ‘good mobility’. Functional ability was evaluated with a question about difficulties in usual activities (e.g., work, family, or leisure activities), also part of the EuroQol 5D-5L questionnaire [21]. A response “I have no problems doing my usual activities” was perceived as ‘good functional ability’. Self-rated health was evaluated with the question “What is your perception of your health in general?” (5-point scale from “very poor” to “excellent”); the responses “good” and “excellent” were perceived as ‘good self-rated health’. Finally, the number of prescribed medications and smoking history were queried at the baseline visit.

Dietary intake was assessed with a combination of a 3-day food record and 24-h recalls in three phases: baseline, 3-month follow-up, and 6-month follow-up. The food records were obtained via telephone interview by a trained nutritionist after each food record day to assess nutrient intake as accurately as possible. Nutrient intake was analyzed using national food composition databases in Finland [22] and The Netherlands [23]. The daily intakes of energy (kcal), fat (g), carbohydrates (including sugars and starch) (g), protein (g and g/aBW), sugars (i.e., mono- and disaccharides) (g), and dietary fiber (g) were calculated. Change in nutrient intake was evaluated between baseline and the 6-month follow-up or between baseline and the 3-month follow-up if the participant had not provided food record data at the 6-month follow-up.

The outcome measures of this secondary analysis are changes in macronutrient intake (i.e., protein, carbohydrates, and fat) calculated in grams (g). We also report the change (g) in the intakes of saturated fat, sugars (i.e. mono- and disaccharides), and dietary fiber. In addition, we evaluate whether the increase in energy intake is associated with changes in macronutrient intakes and relative weight change. Relative weight change was calculated between measured weight at baseline and the 6-month follow-up.

### Statistical analysis

The results are presented as means with standard deviations (SDs) (continuous variables), counts (*n*) with proportions (%), and 95% confidence intervals (CIs). The control and intervention groups were compared using the t test or permutation test for continuous variables, and Chi-square test or Fisher–Freeman test for categorical variables. Repeated measures of the changes in macronutrient intake were compared between the groups with mixed-effects models and an unstructured covariance structure (i.e., Kenward–Roger method for calculating df) [24]. Fixed effects included group (control and intervention groups), time (baseline, 3 months, and 6 months), and group × time interactions. Mixed models allowed the analyses of unbalanced datasets without imputation; therefore, we analyzed all available data with the full analysis set. Assumptions of normal distributions were evaluated graphically and with the Shapiro–Wilk test. We calculated the correlation coefficients using Pearson’s method. All analyses were performed with Stata 17.0 (Stata-Corp LP; College Station, TX, USA).

## Results

There were no differences in baseline characteristics between the control group and the intervention groups (Table 1). Mean age was 75 years (*SD* 4.0 and 5.0), and about half of the participants in both groups were women. Most of the participants had a higher education and were living in cohabitation. Mobility, physical functioning, and self-rated health were considered to be ‘good’.

**Table 1:** Baseline characteristics of study participants by study group

	Control group *n* = 85	Intervention group *n* = 175	*P* value
Women, *n* (%)	47 (55)	91 (52)	0.62
Age, mean (SD)	75 (4)	75 (5)	0.98
Weight, mean (SD)	78 (10)	77 (11)	0.40
Women	74 (8)	71 (8)	0.084
Men	84 (10)	84 (11)	0.79
BMI, mean (SD)	27.0 (2.8)	26.5 (2.8)	0.18
Education, *n* (%)			0.073
Low	12 (14)	21 (12)	
Middle	15 (18)	15 (9)	
High	58 (68)	139 (79)	
Living in cohabitation, *n* (%)	50 (59)	124 (71)	0.053
FAT%, mean (SD)			
Women	38.6 (4.1)	38.1 (4.4)	0.57
Men	27.2 (3.6)	26.3 (5.1)	0.33
Good mobility, *n* (%)	79 (93)	168 (96)	0.29
Good functional ability, *n* (%)	76 (89)	158 (90)	0.83
Good SRH, *n* (%)	68 (80)	136 (78)	0.67
Medicine count, mean (SD)	3.5 (2.9)	3.1 (2.9)	0.40
Current smoker, *n* (%)	3 (4)	4 (2)	0.69

Data are *n* (%) or mean (SD). *P* values are calculated with Fisher Chi permu *t* test

*BMI* body mass index, *FAT%* body fat percentage, *SRH* self-rated health

In addition, the macronutrient intake at baseline was similar in both groups (Table 2). Mean fat intake was 65.6 (*SD* 17.9) g in the control group and 66.4 (*SD* 20.8) g in the intervention groups. Mean carbohydrate intake was 169 (*SD* 46) g in the control group and 173 (*SD* 51) g in the intervention groups. Protein intake was similar in both groups [60.6 (*SD* 10.9) g in control group, and 60.6 (*SD* 12.5) g in intervention groups].

**Table 2:** Change in macronutrient intake and intake of saturated fat, sugars (i.e., mono- and disaccharides), and fiber during the 6-month intervention by study group in the PROMISS trial

	Baseline		Change at 6 months		*P* value
	Control Mean (SD)	Intervention Mean (SD)	Control Mean (95% CI)	Intervention Mean (95% CI)	
Fat, g	65.6 (17.9)	66.4 (20.8)	3.1 (− 1.5 to 7.7)	7.4 (4.2–10.6)	0.13
Saturated fat, g	23.8 (8.5)	24.0 (9.4)	1.6 (− 0.5 to 3.6)	3.4 (2.0–4.8)	0.16
Carbohydrates, g	169 (46)	173 (51)	− 1.3 (− 9.7 to 7.2)	9.5 (3.6–15.4)	0.041
Sugars, g	80.4 (33.3)	81.3 (30.5)	− 3.1 (− 8.7 to 2.5)	2.9 (− 0.9 to 6.8)	0.083
Fiber, g	21.2 (10.8)	20.7 (6.5)	− 0.5 (− 2.0 to 1.0)	0.9 (− 0.2 to 1.9)	0.14
Protein, g	60.6 (10.9)	60.6 (12.5)	2.9 (− 0.9 to 6.6)	28.4 (25.8–31.0)	< 0.001

Data at baseline in grams (SD). Change at 6 months in grams (range of variation). Carbohydrates included starch and sugars. Sugars included digestible carbohydrates, i.e., mono- and disaccharides.

We found a significant increase in the intake of carbohydrates in the intervention groups compared with the control group (Table 2). No change was observed in the intakes of total fat, saturated fat, sugars (i.e., mono- and disaccharides), and dietary fiber.

**Fig. 2:**
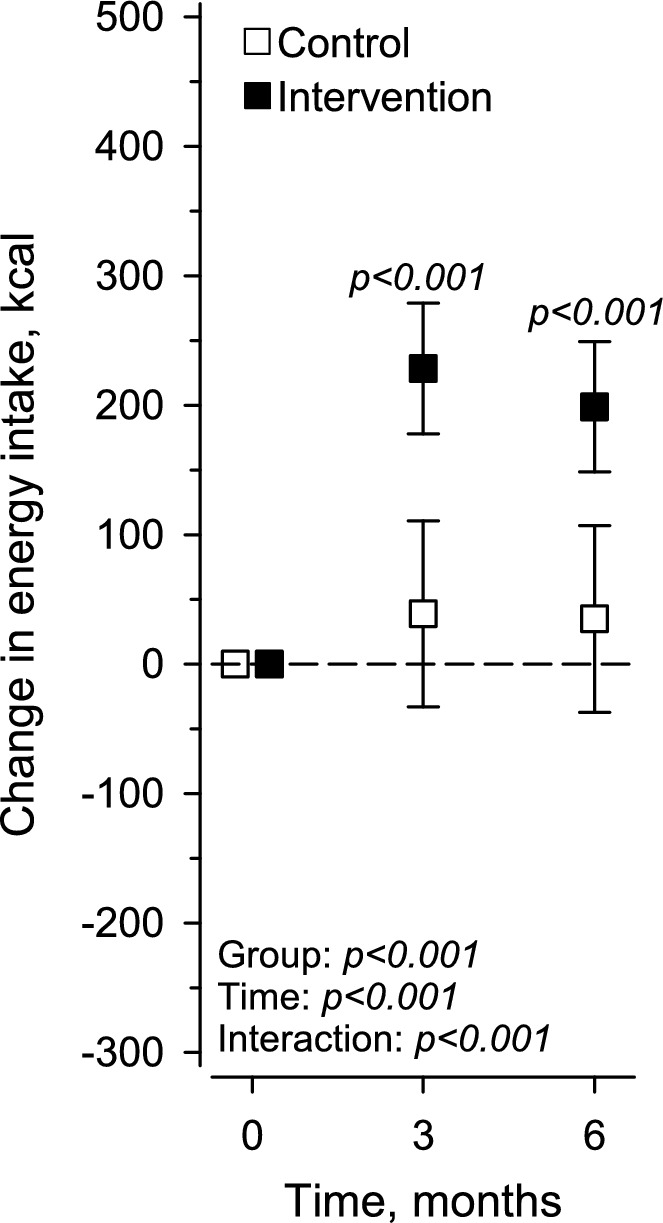
Change in energy intake (kcal) at the 3-month and 6-month follow-ups by study group

During the 6-month intervention, the energy intake increased significantly in the intervention groups compared with the control group [+ 198.89 (*SD* 25.67) kcal and + 34.99 (*SD* 36.86) kcal, respectively] (Fig. 2).

**Fig. 3:**
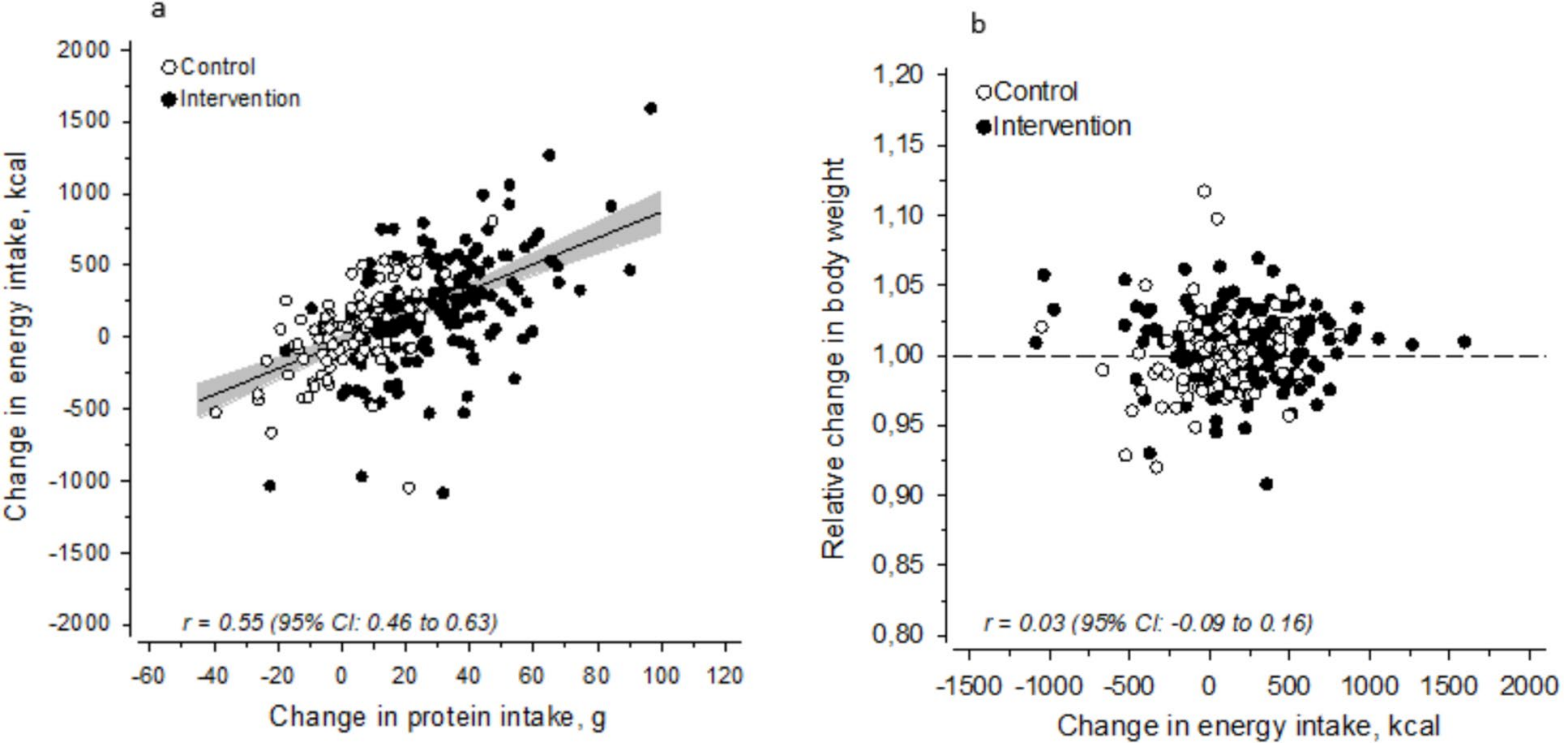
**a** Association between change in protein intake (g) and change in energy intake (kcal) during the 6-month follow-up by study group, **b** association between change in energy intake (kcal) and relative change in body weight during the 6-month follow-up

Change in energy intake was associated with change in protein intake [*r* = 0.55 (95% CI 0.46–0.63)] (Fig. 3A), but not with change in other macronutrients (data not shown). Change in energy intake was not associated with relative weight change during the 6-month intervention (Fig. 3B).

## Discussion

This secondary analysis shows that personalized dietary advice in the PROMISS RCT, which focused only on the intake of protein, increases the intake of carbohydrates (g), in addition to the previously reported increase in the intake of protein [18], among community-dwelling older adults with a low habitual protein intake. Moreover, the increase in energy intake was associated with the increase in protein intake but not with relative change in body weight.

To the best of our knowledge, there are no previous trials exploring how personalized dietary advice aiming only at increasing protein intake changes the intake of other macronutrients among community-dwelling older adults. In many earlier dietary interventions, there have been several goals with varying lengths of follow-up [9, 25–27]. However, it is difficult to compare these intervention studies due to differences in methodologies, target groups, main objectives, and interventions. For example, in a 6-month RCT by Koponen et al. [9] among older Finnish family caregivers, a sole multi-domain dietary advice resulted in an increase in many micronutrients but did not attain the recommended intake of protein. Another trial giving tailored dietary advice for Alzheimer patients’ spousal caregivers did increase protein intake by nutritional supplements [26].

The nutrient intake among the PROMISS study group at baseline reflects the national intake levels found in populational studies in Finland [28] and The Netherlands [29]. However, in Finland, no nutrient intake data exist for a representative sample of people aged over 74 years. The intakes of total fat, saturated fat, and sugars were higher, while the intake of dietary fiber was lower than in the current Nordic Nutrition Recommendations (NNR) published in 2023 [7]. In contrast, the intakes of carbohydrates and energy were lower than recommended levels.

Baseline intake levels enable us to evaluate the clinical significance of the results. It was previously reported that the PROMISS dietary intervention had clinically significant positive results on protein intake [18]. In our secondary analysis, we found a significant increase in carbohydrate intake but no significant changes in the intakes of total fat, saturated fat, sugars (i.e., mono- and disaccharides), and fiber. The carbohydrate intake at baseline was below the recommended level [7]. The observed increase of 9.5 g (95% CI 3.6–15.4) is statistically—but not clinically—significant. The small increase might be due to the provided protein-enriched food products (e.g., puddings, protein bar, whole‐grain cereals, whey protein powder, and protein drink), which contained small amounts of carbohydrates.

Since the original aim of the PROMISS RCT was to keep the dietary advice energy neutral, we further analyzed the previously reported increase in energy intake. Our secondary analysis shows that the increase in energy intake was associated with the change in protein intake, which might indicate that the participants simply increased food intake instead of replacing low protein foods with higher protein alternatives. Based on food records, many of the participants were eating fairly small amounts of food at the beginning of the study, and some were as a result struggling to increase their protein intake [19]. Possibly, our results may be explained as an underreporting of food intake at baseline and/or an overreporting of intake at follow-up.

As obesity is becoming more prevalent among older adults [30], sarcopenia is now more often accompanied by obesity. A recent systematic review and meta-analysis [14] including 50 studies around the world showed that more than one out of ten older adults globally have sarcopenic obesity. While adequate protein intake has multiple benefits on sarcopenia [1], dietary interventions should not induce excess energy intake that might lead to increase in body weight. In this study, increase in energy intake did not lead to increase in body weight; however, this might be explained by under-/overreporting of food intake as discussed above, or by that change in body weight might require a longer follow-uptime.

Our secondary analysis has several strengths but also some limitations. One significant strength is the RCT setting. The participants in the intervention groups received personalized dietary advice based on their usual diet, which has been shown to be more effective than generalized dietary advice in the previous studies [31]. In addition, participants were given written instructions and contacted monthly by phone to enhance compliance to the diet. We used a sample of Dutch and Finnish participants, creating a culturally heterogeneous sample of community-dwelling older adults. Furthermore, food intake was evaluated with a combination of a 3-day food record and 24-h recalls performed by trained nutritionists, which minimized the chances of misreporting due to memory limitations. However, we must acknowledge the likelihood of misreporting when using dietary intake data based on self-reporting [32]; this is a clear limitation of the study.

There are other limitations that should be considered. Our trial is not comparable to the results of dietary intervention studies aimed at increasing protein intake without using protein-enriched food products. Furthermore, the participants in the PROMISS RCT were highly educated, which is associated with healthier food choices [33]. Thus, our study sample might not be representative of the general older community-dwelling population.

## Conclusions

This secondary analysis shows that dietary advice to increase protein intake also affects intake of other macronutrients, i.e., carbohydrates; however, the intakes of dietary fiber, sugars, and (saturated) fat remained at baseline levels. Energy intake increased alongside the increase in protein intake. Interestingly, this was not reflected in a change in body weight.

## Data Availability

The data that support the findings of this study are available from the corresponding author upon reasonable request.
